# Depression among frontline medical and paramedical staff during the coronavirus pandemic

**DOI:** 10.1192/j.eurpsy.2022.1341

**Published:** 2022-09-01

**Authors:** O. Maatouk, R. Kammoun, I. Kammoun, K. Souabni, M. Karoui, H. Nefzi, F. Ellouz

**Affiliations:** 1 Razi Hospital, F Adult Psychiatry Department, Manouba, Tunisia; 2 Razi hospital, Psychiatry G, manouba, Tunisia; 3 Razi Hospital, Avicenne Psychiatry Department, Mannouba, Tunisia; 4 Razi Hospital, Psychiatry D, Manouba, Tunisia; 5 Razi hospital, Psychiatry G Department, Mannouba, Tunisia; 6 Razi hospital, Psychiatry G, denden, Tunisia

**Keywords:** Depression, frontline staff, Coronavirus-2019

## Abstract

**Introduction:**

The current coronavirus pandemic is a unique and unusual situation. It is putting the general population under severe strain. However, frontline medical and paramedical staff remain particularly vulnerable to depression because of its close contact with patients.

**Objectives:**

The aim of this work was to screen and evaluate depression in the frontline professionals during the pandemic and to study their associated factors .

**Methods:**

In this study , we conducted a national descriptive and analytical cross-sectional study over a 2-month period from September to October 2020. We used “Beck Depression Inventory” to assess depression and “Brief Cope Scale” to detect a possible correlation between depression and coping mechanisms.

**Results:**

We collected 78 professionals. The mean age was 29.86 years. 2/3 of workers were women. 67.9% of the staff were residents. 39.7% worked in Covid units. 7.7% had personal psychiatric history. 56.4% of the staff worked daily and 76.9% of them provided direct care to patients with Coronavirus. 52.6% of workers did not receive adequate training of protection against Covid-19.The staff reported 66.7% of death among their patients. 42.3% suffered from minor depression and only 2.3% suffered from severe depression. During this period we objectified an increase of 14.1% in the psychoactive substances use. Stigma affected 57.7% of professionals. We didn’t objectify a significant correlation between Depression and coping mechanisms .

**Conclusions:**

Screening depression among healthcare professionals should be considered in order to prevent it, ensure continuity of care and avoid sick leaves.

**Disclosure:**

No significant relationships.

